# Longitudinal Characterization of *Escherichia coli* in Healthy Captive Non-Human Primates

**DOI:** 10.3389/fvets.2014.00024

**Published:** 2014-11-17

**Authors:** Jonathan B. Clayton, Jessica L. Danzeisen, Ava M. Trent, Tami Murphy, Timothy J. Johnson

**Affiliations:** ^1^Department of Veterinary and Biomedical Sciences, College of Veterinary Medicine, University of Minnesota, Saint Paul, MN, USA; ^2^Department of Veterinary Population Medicine, College of Veterinary Medicine, University of Minnesota, Saint Paul, MN, USA; ^3^Como Park Zoo & Conservatory, Saint Paul, MN, USA

**Keywords:** *Escherichia coli*, virulence, plasmid, antibiotic resistance, non-human primate

## Abstract

The gastrointestinal (GI) tracts of non-human primates (NHPs) are well known to harbor *Escherichia coli*, a known commensal of human beings and animals. While *E. coli* is a normal inhabitant of the mammalian gut, it also exists in a number of pathogenic forms or pathotypes, including those with predisposition for the GI tract as well as the urogenital tract. Diarrhea in captive NHPs has long been a problem in both zoo settings and research colonies, including the Como Zoo. It is an animal welfare concern, as well as a public health concern. *E. coli* has not been extensively studied; therefore, a study was performed during the summer of 2009 in collaboration with a zoo in Saint Paul, MN, which was previously experiencing an increased incidence and severity of diarrhea among their NHP collection. Fresh fecal samples were collected weekly from each member of the primate collection, between June and August of 2009, and *E. coli* were isolated. A total of 33 individuals were included in the study, representing eight species. *E. coli* isolates were examined for their genetic relatedness, phylogenetic relationships, plasmid replicon types, virulence gene profiles, and antimicrobial susceptibility profiles. A number of isolates were identified containing virulence genes commonly found in several different *E. coli* pathotypes, and there was evidence of clonal transmission of isolates between animals and over time. Overall, the manifestation of chronic diarrhea in the Como Zoo primate collection is a complex problem whose solution will require regular screening for microbial agents and consideration of environmental causes. This study provides some insight toward the sharing of enteric bacteria between such animals.

## Introduction

*Escherichia coli* is a Gram-negative bacteria and known gut commensal of animals, including non-human primates (NHPs) (1–11). This diverse organism not only plays a role in the maintenance of gut health by helping to prevent the establishment of pathogenic bacteria in the gastrointestinal (GI) tract, but can also exist in a number of pathogenic forms (5, 12). In fact, pathogenic forms of *E. coli* are a major cause of diarrheal illness worldwide (13, 14). In addition, *E. coli* is a model laboratory organism, which has been used to examine basic evolutionary processes, bacterial pathogenesis, and epidemiological transmission (14–17). The ubiquity and ease of isolation of *E. coli* have enabled ecological studies to investigate how anthropogenic change (e.g., forest fragmentation) affects bacterial transmission among wildlife, including primates, human beings, and livestock (18–21). Information generated from these types of studies provides insight into the disease transmission process, enabling for the calculation of potential risk factors associated with human encroachment on wildlife populations and vice versa (18–21). Such studies also aid in the development and implementation of conservation strategies to protect endangered wildlife (18–21).

Diarrhea in captive primates has long been a problem in both zoo settings and research colonies (22–28). In fact, since at least 1998, the primate collection at the Como Zoo in Saint Paul, MN, has experienced issues with diarrhea. Diarrhea is a medical concern not only because it is physiologically taxing to the animals, but also because it is an ideal mechanism for disease transmission, making it both a public health concern and an animal welfare concern. Management of intermittent diarrhea is confounded by its complexities, including the lack of a consistently identifiable causative agent.

In many animals, including primates, some pathotypes of *E. coli* such as enteropathogenic *E. coli* (EPEC) are associated with the development of persistent diarrhea (3, 7, 29). Although the *E. coli* pathotypes have been clearly defined, they often differ in virulence between hosts, even between closely related mammals (21, 30, 31). For example, one of the most pathogenic forms of *E. coli* toward human beings is Shiga toxin-producing *E. coli*, or STEC, which includes serotype 0157:H7. STEC are commensal organisms in ruminants, but are highly virulent pathogens in human beings that can cause severe hemorrhagic colitis (32–34). In NHPs, it is unclear whether certain pathotypes exist as commensals or pathogens.

In this study, we examined *E. coli* isolates from 33 NHPs within a single zoo over a 7-week period, surveying genetic relatedness using a sequence-based approach, phylogenetic types, plasmid replicon types, virulence gene profiles, and antimicrobial susceptibility profiles. *E. coli* was the organism most suited for the purposes of this study, as it is easily culturable, has been previously implicated in episodes of diarrheal illness in captive primate populations, and is an ideal organism to assess genetic relatedness and microbial gene flow. The goal of this work was to determine whether primates are in fact a reservoir for *E. coli* harboring virulence genes and plasmid types typical of different *E. coli* pathotypes, to determine the genetic relatedness of *E. coli* over time and between co-habitating (i.e., same primate complex) animal groups, to assess the antimicrobial susceptibilities of *E. coli* isolates, and to evaluate the potential link between the zoo’s history of diarrhea with pathogenic *E. coli* presence.

## Materials and Methods

### Primate study population and fecal sample collection

The research in this study complied with protocols approved through the University of Minnesota Institutional Animal Care and Use Committee. The primate collection at the Como Zoo in Saint Paul, MN, an American Zoological Association (AZA) accredited zoo, served as the study population, which included 33 healthy primates representing eight primate species (Table 1). Fresh fecal samples were collected weekly from each primate, between June and August of 2009. Samples were collected from freshly voided feces using a sterile spatula, with care to collect from the top of the sample to avoid ground contamination. After collection, samples were immediately placed on ice for transport from the zoo to the laboratory and subsequently processed.

**Table 1:** **Primate populations included in this study**.

Animal ID	Common name	Scientific name	Sex	Age (years, as of 2009)
WLG1	Western lowland gorilla	*G. gorilla gorilla*	Male	24
WLG2	Western lowland gorilla	*G. gorilla gorilla*	Male	23
WLG3	Western lowland gorilla	*G. gorilla gorilla*	Male	21
O1	Orangutan	*P. sp. hybrid*	Female	33
O2	Orangutan	*P. abelii*	Male	24
O3	Orangutan	*P. abelii*	Female	22
O4	Orangutan	*P. abelii*	Male	2
DM1	De Brazza’s monkey	*C. neglectus*	Female	12
DM2	De Brazza’s monkey	*C. neglectus*	Male	11
BHSM1	Black-handed spider monkey	*A. geoffroyi*	Male	19
BHSM2	Black-handed spider monkey	*A. geoffroyi*	Male	18
BHSM3	Black-handed spider monkey	*A. geoffroyi*	Female	18
BHSM4	Black-handed spider monkey	*A. geoffroyi*	Female	18
BHSM5	Black-handed spider monkey	*A. geoffroyi*	Female	13
WFS1	White-faced saki	*P. pithecia*	Male	9
WFS2	White-faced saki	*P. pithecia*	Female	11
WFS3	White-faced saki	*P. pithecia*	Male	4
WFS4	White-faced saki	*P. pithecia*	Male	3
WFS5	White-faced saki	*P. pithecia*	Female	1
BEBL1	Blue-eyed black lemur	*E. macaco flavifrons*	Male	15
BEBL2	Blue-eyed black lemur	*E. macaco flavifrons*	Female	12
ET1	Emperor tamarin	*S. imperator subgrisescens*	Male	21
ET2	Emperor tamarin	*S. imperator subgrisescens*	Female	11
ET3	Emperor tamarin	*S. imperator subgrisescens*	Male	2
ET4	Emperor tamarin	*S. imperator subgrisescens*	Female	2
ET5	Emperor tamarin	*S. imperator subgrisescens*	Male	2
ET6	Emperor tamarin	*S. imperator subgrisescens*	Male	2
ET7	Emperor tamarin	*S. imperator subgrisescens*	Male	17
ET8	Emperor tamarin	*S. imperator subgrisescens*	Female	12
ET9	Emperor tamarin	*S. imperator subgrisescens*	Male	3
ET10	Emperor tamarin	*S. imperator subgrisescens*	Male	3
GT1	Geoffroy’s tamarin	*S. geoffroyi*	Male	12
GT2	Geoffroy’s tamarin	*S. geoffroyi*	Female	9

*G.: Gorilla; P.: Pongo; C.: Cercopithecus; A.: Ateles; P.: Pithecia; E.: Eulemur; S.: Saguinus*.

### Bacterial isolates

*E. coli* isolates (*N* = 162) were included in this study. *E. coli* was isolated from fecal samples as follows: 1 g of fresh fecal material was homogenized in 10 mL of buffered peptone water (BD, Sparks, MD, USA). Ten-fold serial dilutions were performed on each sample, and dilutions were plated on MacConkey agar (BD). Plates with isolated colonies were used to pick one presumptive colony per fecal sample. Suspect *E. coli* colonies were then re-plated on Levine eosin methylene blue (EMB) agar (BD) as a secondary confirmation of *E. coli*. Finally, the suspect *E. coli* colonies from the EMB agar were confirmed using PCR primers specific for *E. coli* (35).

### DNA extraction and PCR analysis

DNA templates for PCR were prepared using boiled lysates of overnight cultures from each individual sample being examined (36). A total of 22 *E. coli* control strains were used in this study (Table S1 in Supplementary Material). For plasmid replicon typing and virulence gene screening, amplifications were performed in a 25 μL reaction mixture, containing 5 μL 5 × PCR buffer (Promega, Madison, WI, USA), 0.25 mM dNTP mixture, 2.5 U *Taq* DNA polymerase (Promega), 0.15 μmol of each primer, and 2 μL template DNA. Following PCR (described below), the samples were subjected to horizontal gel electrophoresis in a 2.0% (w/v) Tris–acetate–EDTA (TAE) agarose, and the size of the amplicons was determined by comparison to the 100 bp DNA marker and known positive controls (New England Biolabs, Beverly, MA, USA).

### Phylogenetic group typing

Isolates were assigned to one of the four phylogenetic groups (A, B1, B2, or D) using the interpretive approach of Clermont et al. (37). As determined by PCR, isolates were assigned to groups based on their possession of two genes (*chuA* and *yjaA*) and a DNA fragment (TSPE4.C2).

### Sequence-based typing

A subset of isolates was examined for genetic relatedness using *fumC* gene sequencing, as previously described (38). For each isolate, PCR amplicons were generated and subjected to bidirectional sequencing using the same PCR primers used for amplification. Resulting sequences were quality trimmed, assembled, and concatenated to a similar size. Phylogenetic relationships between the isolates were examined using MEGA5 (39). Data from this project are freely available at Figshare (http://figshare.com/articles/Zoo_E_coli_fumC_dataset/956237).

### Virulence genotyping

Test and control organisms were examined for the presence of 10 genes known for their association with Extraintestinal pathogenic *E. coli* (ExPEC) virulence: *cvaC*, *etsB, hlyA, hlyF, ireA, iroN, iss, iutA, papC*, and *sitA* (40, 41); three genes associated with EPEC virulence: *eae, espC*, and *tir*; three genes associated with Enterotoxigenic *E. coli* (ETEC) virulence: *eltA, sta*, and *stb*; three genes associated with Enterohemorrhagic *E. coli* (EHEC) virulence: *etpD, stx1A*, and *stx2A*; three genes associated with Enteroaggregative *E. coli* (EAEC) virulence: *aatP, aggR*, and *pet;* and two genes associated with Enteroinvasive *E. coli* (EIEC) virulence: *sepA* and *shET2*. The multiplex procedures for the majority of ExPEC-associated genes screened for in this study are previously described (40). Three additional multiplex PCR assays were designed in this study to screen for the remainder of genes (Table S2 and Figure S1 in Supplementary Material). Reaction mixtures associated with the three multiplex PCR assays designed in this study were subjected to the following cycling parameters: initial denaturation (95°C, 10 min), followed by 30 cycles of denaturation (94°C, 30 s), annealing (55°C, 30 s), and extension (72°C, 60 s), and a final extension (72°C, 7 min). Specificity of the primers was confirmed using known positive and negative bacterial controls.

### Plasmid replicon typing

Isolates were examined for the presence of 15 sequences specific for different plasmid incompatibility types using a combination of multiplex PCR assays previously described (42). The following plasmid incompatibility groups were sought: IncP-1α, IncA/C, IncFIC, IncB/O, IncK/B, IncW, IncFIA, IncFIB, IncY, IncI1, IncX, IncHI1, IncN, IncHI2, and IncL/M.

### Antimicrobial susceptibility testing

*E. coli* isolates (*N* = 162) were tested for susceptibility to seven antimicrobial agents by Kirby-Bauer disk diffusion, according to the Clinical Laboratory Standards Institute (CLSI) MIC Interpretative Standards using Mueller–Hinton agar plates (43, 44). Disks containing the following antimicrobial agents were used: ampicillin (AM, 10 μg), gentamicin (GM, 10 μg), nalidixic acid (NA, 30 μg), streptomycin (S, 10 μg), tetracycline (TE, 30 μg), sulfisoxazole (G, 0.25 mg) and trimethoprim (TMPS or W5, 5 μg) (BD). The following *E. coli* strains were used as positive controls: ATCC 25922 and APEC 01 (43–45).

## Results

Over the course of this seven-week study, a total of 229 fecal samples were collected from the Como Zoo’s primate collection. Of the 229 fecal samples collected, *E. coli* was successfully isolated from 162 samples (70.7%). Unsuccessful isolations typically involved overgrowth with *Proteus* spp. which was unable to be resolved. For the *E. coli* isolates (*N* = 162) used in this study, one colony per sample was selected. The majority of the individuals were sampled weekly throughout the seven-week study period (i.e., seven total samples collected per individual). However, there were two instances where an individual was not sampled due to unavailable sample material during the designated sample collection period.

### Phylogenetic group typing

Phylogenetic groups A, B1, B2, and D were all found to be present in the captive population of NHPs tested in this study. Of the four phylogenetic groups tested for in this study, B1 was the most commonly identified group, with 53.1% (*N* = 86) of the isolates testing positive. In addition, 22.8% (*N* = 37) of the isolates tested positive for group A and 19.8% (*N* = 32) of the isolates tested positive for group B2. The least prevalent phylogenetic group in this study was group D, which tested positive in 4.3% (*N* = 7) of the isolates.

### Sequence-based typing

**Figure 1:**
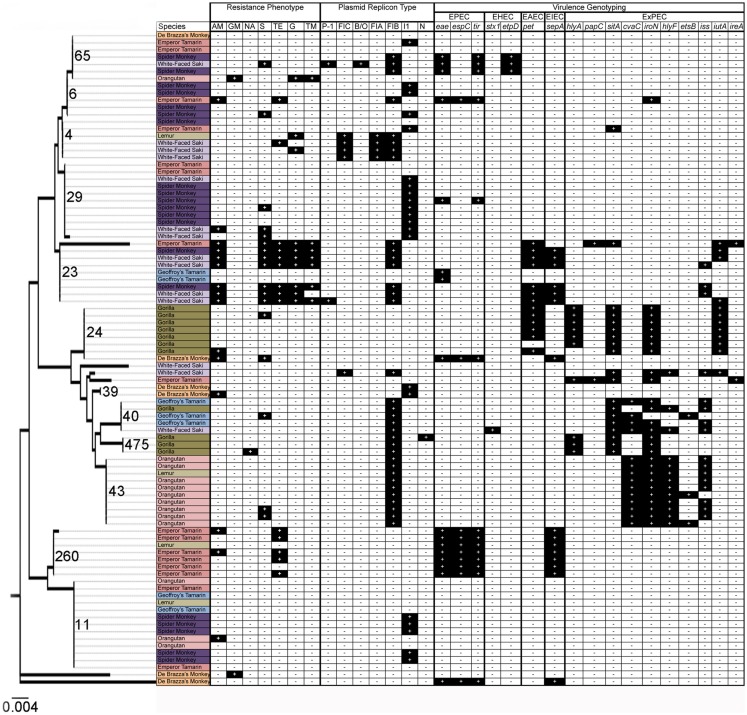
**Results of resistance phenotyping, plasmid replicon analysis, virulence genotyping, and *fumC* sequence analysis among 91 isolates in this study**. Species are colored as follows: orange: De Brazza’s monkey; light pink: orangutan; pink: emperor tamarin; dark purple: spider monkey; light purple: white-faced saki; light green: lemur; dark green: gorilla; and light blue: Geoffroy’s tamarin. Dendrogram depicts relationships of isolates using *fumC* sequence analysis.

Sequence-based typing was performed on a subset (*N* = 91) of the *E. coli* isolates, selected to be representative of the total population based upon date of isolation and animal source, using the *fumC* gene (Figure 1). A total of 18 *fumC* sequence types were identified using the Achtman *E. coli* MLST database (46). Sequence types spanned multiple isolates, species, and collection time points. Some common patterns were observable, though. For example, in many cases isolates were identified that originated from the same animal at different time points, and possessed identical sequence types as well as identical genotyping profiles, suggesting common clones persisting over time. There was also evidence of transfer of common *E. coli* clones between animals of the same species, and between animals of differing species over time. In particular, the following identical genotyping patterns were observed: *fumC*65 (two sets of two isolates), *fumC*29 (five isolates), *fumC*23 (two sets of two isolates), *fumC*24 (four isolates), *fumC*43 (eight isolates), *fumC*260 (three sets of two isolates), and *fumC*11 (two sets of seven and five isolates).

### Plasmid replicon typing

All eight NHP species examined were found to harbor *E. coli* with DNA sequences associated with at least one of the 15 plasmid incompatibility groups sought in this study. Of the 15 plasmid incompatibility groups examined, 9 were found to be present in the isolates, including IncFIB, IncI1, IncFIC, IncP-1α, IncB/O, IncFIA, IncK/B, IncN, and IncW (Table 2). However, only two plasmid types were prevalent among isolates at rates greater than 10% (RepFIB and RepI1).

**Table 2:** **Overall prevalence of each gene and plasmid replicon studied (at least one positive isolate)**.

Gene/Replicon	Prevalence (%) (*N* = 162)
IncP-1α	6.8 (*N* = 11)
IncFIC	8.0 (*N* = 13)
IncB/O	3.7 (*N* = 6)
IncK/B	2.5 (*N* = 4)
IncW	0.6 (*N* = 1)
IncFIA	3.1 (*N* = 5)
IncFIB	25.3 (*N* = 41)
IncI1	14.2 (*N* = 23)
IncN	1.2 (*N* = 2)
*hlyA*	7.4 (*N* = 12)
*papC*	1.9 (*N* = 3)
*sitA*	19.8 (*N* = 32)
*cvaC*	8.6 (*N* = 14)
*iroN*	17.3 (*N* = 28)
*hlyF*	8.0 (*N* = 13)
*etsB*	1.9 (*N* = 3)
*iss*	10.5 (*N* = 17)
*aerJ* (*iutA*)	8.0 (*N* = 13)
*ireA*	1.9 (*N* = 3)
*eae*	13.0 (*N* = 21)
*espC*	8.0 (*N* = 13)
*tir*	9.3 (*N* = 15)
*eltA*	0.6 (*N* = 1)
*sta*	0.6 (*N* = 1)
*pet*	9.3 (*N* = 15)
*stx1A*	4.9 (*N* = 8)
*etpD*	2.5 (*N* = 4)
*sepA*	10.5 (*N* = 17)
*shET2*	1.2 (*N* = 2)

### Virulence genotyping

Screening for 24 virulence factors associated with ExPEC, EPEC, ETEC, EAEC, EHEC, and EIEC, all eight NHP species tested were found to contain *E. coli* possessing known virulence factors. Of these 24 virulence factors, 20 were identified in the *E. coli* isolates from 31 of the 33 primates in this study.

Of the 10 ExPEC-associated genes sought, all were found to be present in at least one NHP, including *sitA*, *iroN, iss*, *cvaC*, *hlyF*, *iutA*, *hlyA*, *etsB*, *ireA*, and *papC* (Table 2). Furthermore, ExPEC-associated genes were found among *E. coli* isolated from every NHP species examined (Table 3). In terms of gene function, ExPEC genes sought included those associated with iron acquisition (*sitA*, *iroN*, *iutA*, *ireA*, and *etsB*), serum survival (*iss*), colicin production (*cvaC*), hemolysin production (*hlyA* and *hlyF*), and fimbriae production (*papC*) (40, 41, 47–50). Three ExPEC-associated genes were present in greater than 10% of the isolates examined: *sitA* (19.8%), *iroN* (17.3%), and *iss* (10.5%). These genes often occurred together in the same isolates. At weeks 6 and 7 of sampling, ExPEC virulence genes had the highest prevalence of all genes screened (Table 4).

**Table 3:** **Prevalence of each gene and plasmid replicon studied by primate species**.

Gene/Replicon	Gorilla	Orangutan	De Brazza’s Monkey	Spider Monkey	White-faced Saki	Lemur	Emperor Tamarin	Geoffroy’s Tamarin
**Prevalence of genes screened by primate species (at least one positive isolate)**								
IncP-1α	10.5% (2)	14.8% (4)	0.0% (0)	0.0% (0)	11.1% (3)	0.0% (0)	6.5% (2)	0.0% (0)
IncFIC	5.3% (1)	0.0% (0)	16.7% (2)	0.0% (0)	18.5% (5)	20.0% (1)	16.1% (5)	0.0% (0)
IncB/O	5.3% (1)	0.0% (0)	0.0% (0)	2.9% (1)	7.4% (2)	0.0% (0)	6.5% (2)	0.0% (0)
IncK/B	10.5% (2)	3.7% (1)	0.0% (0)	2.9% (1)	0.0% (0)	0.0% (0)	0.0% (0)	0.0% (0)
IncW	0.0% (0)	0.0% (0)	0.0% (0)	0.0% (0)	0.0% (0)	0.0% (0)	3.2% (1)	0.0% (0)
IncFIA	0.0% (0)	0.0% (0)	0.0% (0)	0.0% (0)	14.8% (4)	20.0% (1)	0.0% (0)	0.0% (0)
IncFIB	21.1% (4)	37.0% (10)	0.0% (0)	17.6% (6)	48.1% (13)	40.0% (2)	9.7% (3)	42.9% (3)
IncI1	0.0% (0)	0.0% (0)	33.3% (4)	44.1% (15)	11.1% (3)	0.0% (0)	9.7% (3)	0.0% (0)
IncN	5.3% (1)	3.7% (1)	0.0% (0)	0.0% (0)	0.0% (0)	0.0% (0)	0.0% (0)	0.0% (0)
*hlyA*	47.4% (9)	3.7% (1)	0.0% (0)	2.9% (1)	0.0% (0)	0.0% (0)	3.2% (1)	0.0% (0)
*papC*	0.0% (0)	0.0% (0)	0.0% (0)	0.0% (0)	0.0% (0)	0.0% (0)	9.7% (3)	0.0% (0)
*sitA*	57.9% (11)	22.2% (6)	8.3% (1)	14.7% (5)	7.4% (2)	0.0% (0)	12.9% (4)	42.9% (3)
*cvaC*	0.0% (0)	33.3% (9)	0.0% (0)	0.0% (0)	3.7% (1)	20.0% (1)	0.0% (0)	42.9% (3)
*iroN2*	57.9% (11)	37.0% (10)	0.0% (0)	0.0% (0)	7.4% (2)	20.0% (1)	9.7% (3)	28.6% (2)
*hlyF2*	5.3% (1)	33.3% (9)	0.0% (0)	0.0% (0)	7.4% (2)	20.0% (1)	0.0% (0)	0.0% (0)
*etsB*	0.0% (0)	7.4% (2)	0.0% (0)	0.0% (0)	0.0% (0)	0.0% (0)	0.0% (0)	14.3% (1)
*iss*	5.3% (1)	25.9% (7)	0.0% (0)	2.9% (1)	14.8% (4)	20.0% (1)	3.2% (1)	28.6% (2)
*iutA*	36.8% (7)	0.0% (0)	0.0% (0)	2.9% (1)	14.8% (4)	0.0% (0)	3.2% (1)	0.0% (0)
*ireA*	0.0% (0)	0.0% (0)	0.0% (0)	0.0% (0)	0.0% (0)	0.0% (0)	9.7% (3)	0.0% (0)
*eae*	0.0% (0)	0.0% (0)	25.0% (3)	11.8% (4)	11.1% (3)	20.0% (1)	29.0% (9)	28.6% (2)
*espC*	0.0% (0)	0.0% (0)	25.0% (3)	0.0% (0)	3.7% (1)	20.0% (1)	29.0% (9)	0.0% (0)
*tir*	0.0% (0)	0.0% (0)	25.0% (3)	8.8% (3)	3.7% (1)	20.0% (1)	25.8% (8)	0.0% (0)
*eltA*	0.0% (0)	0.0% (0)	0.0% (0)	0.0% (0)	0.0% (0)	0.0% (0)	3.2% (1)	0.0% (0)
*sta*	0.0% (0)	0.0% (0)	8.3% (1)	0.0% (0)	0.0% (0)	0.0% (0)	0.0% (0)	0.0% (0)
*pet*	31.6% (6)	0.0% (0)	0.0% (0)	5.9% (2)	18.5% (5)	0.0% (0)	6.5% (2)	0.0% (0)
*stx1A*	0.0% (0)	0.0% (0)	25.0% (3)	2.9% (1)	11.1% (3)	0.0% (0)	6.5% (2)	0.0% (0)
*etpD*	0.0% (0)	0.0% (0)	0.0% (0)	8.8% (3)	3.7% (1)	0.0% (0)	0.0% (0)	0.0% (0)
*sepA*	0.0% (0)	0.0% (0)	25.0% (3)	5.9% (2)	18.5% (5)	20.0% (1)	22.6% (7)	0.0% (0)
*shET2*	0.0% (0)	0.0% (0)	16.7% (2)	0.0% (0)	0.0% (0)	0.0% (0)	3.2% (1)	0.0% (0)

**Table 4:** **Prevalence of gene and plasmid replicon among the whole population over time**.

Gene/Replicon	Week 1	Week 2	Week 3	Week 4	Week 5	Week 6	Week 7
**Prevalence of genes and plasmid replicons screened by study week (at least one positive isolate)**							
IncP-1α	0.0% (0)	0.0% (0)	20.8% (5)	0.0% (0)	26.1% (6)	0.0% (0)	0.0% (0)
IncFIC	5.0% (1)	4.8% (1)	8.3% (2)	18.2% (4)	8.7% (2)	3.2% (1)	9.5% (2)
IncB/O	0.0% (0)	0.0% (0)	20.8% (5)	0.0% (0)	4.3% (1)	0.0% (0)	0.0% (0)
IncK/B	0.0% (0)	0.0% (0)	4.2% (1)	0.0% (0)	13.0% (3)	0.0% (0)	0.0% (0)
IncW	5.0% (1)	0.0% (0)	0.0% (0)	0.0% (0)	0.0% (0)	0.0% (0)	0.0% (0)
IncFIA	0.0% (0)	4.8% (1)	4.2% (1)	9.1% (2)	0.0% (0)	3.2% (1)	0.0% (0)
IncFIB	10.0% (2)	42.9% (9)	33.3% (8)	59.1% (13)	17.4% (4)	12.9% (4)	4.8% (1)
IncI1	15.0% (3)	4.8% (1)	12.5% (3)	13.6% (3)	30.4% (7)	9.7% (3)	14.3% (3)
IncN	0.0% (0)	0.0% (0)	4.2% (1)	0.0% (0)	4.3% (1)	0.0% (0)	0.0% (0)
*hlyA*	0.0% (0)	14.3% (3)	16.7% (4)	4.5% (1)	8.7% (2)	6.5% (2)	0.0% (0)
*papC*	0.0% (0)	4.8% (1)	4.2% (1)	4.5% (1)	0.0% (0)	0.0% (0)	0.0% (0)
*sitA*	10.0% (2)	28.6% (6)	29.2% (7)	18.2% (4)	13.0% (3)	12.9% (4)	28.6% (6)
*cvaC*	0.0% (0)	19.0% (4)	8.3% (2)	18.2% (4)	8.7% (2)	6.5% (2)	0.0% (0)
*iroN*	20.0% (4)	28.6% (6)	20.8% (5)	22.7% (5)	17.4% (4)	12.9% (4)	4.8% (1)
*hlyF*	5.0% (1)	14.3% (3)	8.3% (2)	9.1% (2)	8.7% (2)	6.5% (2)	4.8% (1)
*etsB*	0.0% (0)	4.8% (1)	0.0% (0)	9.1% (2)	0.0% (0)	0.0% (0)	0.0% (0)
*iss*	5.0% (1)	14.3% (3)	12.5% (3)	22.7% (5)	8.7% (2)	6.5% (2)	4.8% (1)
*iutA*	5.0% (1)	19.0% (4)	12.5% (3)	4.5% (1)	8.7% (2)	3.2% (1)	4.8% (1)
*ireA*	0.0% (0)	4.8% (1)	4.2% (1)	4.5% (1)	0.0% (0)	0.0% (0)	0.0% (0)
*eae*	20.0% (4)	4.8% (1)	16.7% (4)	9.1% (2)	17.4% (4)	3.2% (1)	23.8% (5)
*espC*	25.0% (5)	0.0% (0)	12.5% (3)	0.0% (0)	13.0% (3)	3.2% (1)	4.8% (1)
*tir*	15.0% (3)	0.0% (0)	16.7% (4)	9.1% (2)	17.4% (4)	3.2% (1)	4.8% (1)
*eltA*	5.0% (1)	0.0% (0)	0.0% (0)	0.0% (0)	0.0% (0)	0.0% (0)	0.0% (0)
*sta*	0.0% (0)	4.8% (1)	0.0% (0)	0.0% (0)	0.0% (0)	0.0% (0)	0.0% (0)
*pet*	5.0% (1)	14.3% (3)	16.7% (4)	18.2% (4)	8.7% (2)	3.2% (1)	0.0% (0)
*stx1A*	5.0% (1)	4.8% (1)	8.3% (2)	0.0% (0)	4.3% (1)	9.7% (3)	0.0% (0)
*etpD*	0.0% (0)	4.8% (1)	4.2% (1)	9.1% (2)	0.0% (0)	0.0% (0)	0.0% (0)
*sepA*	10.0% (2)	9.5% (2)	16.7% (4)	13.6% (3)	17.4% (4)	3.2% (1)	4.8% (1)
*shET2*	0.0% (0)	0.0% (0)	0.0% (0)	0.0% (0)	8.7% (2)	0.0% (0)	0.0% (0)

Of the three EPEC-associated genes screened, all three were present in at least one primate, including *eae* (N = 21), *tir* (N = 15), and *espC* (N = 13) (Table 2). EPEC-associated genes were present in six of eight primate species examined in this study, including Old World monkeys, New World monkeys, and lemurs. Isolates from only two species, gorillas, and orangutans, did not harbor EPEC-associated genes. Both primate species are great apes and belong to the same taxonomic family as humans (i.e., Hominidae) (Table 3). The most prevalent EPEC-associated gene, *eae* (13.0%), which is required for attaching/effacing adherence in *E. coli* (51, 52), was identified in isolates from the following primate species: De Brazza’s monkeys, spider monkeys, white-faced sakis, lemurs, emperor tamarins, and Geoffroy’s tamarins (Table 4). The EPEC-associated gene, *tir* (9.3%), which plays a role in adherence to small intestinal epithelial cells (51), was identified in isolates from the following primate species: De Brazza’s monkeys, spider monkeys, white-faced sakis, lemurs, and emperor tamarins (Table 4). The EPEC-associated gene, *espC* (8.0%), which is responsible for the production of an autotransporter protein/enterotoxin (53), was identified in isolates from the following primate species: De Brazza’s monkeys, white-faced sakis, lemurs, and emperor tamarins (Table 3). The *espC-*encoded protein is unique as it is the only one of the five proteins secreted extracellularly that is secreted independently of the type III secretion system encoded on the enterocyte effacement pathogenicity island (53).

Of the three ETEC-associated genes screened, *sta* and *eltA* were present in at least one NHP albeit at very low prevalence (Table 2), while *stb* was not found in any isolates examined. *sta* and *eltA* are both enterotoxin-encoding genes (54). *sta* encoding heat-stable toxin type A and *eltA* encoding heat-labile enterotoxin A prepeptide gene (54).

Of the three EAEC-associated genes screened, only *pet* was present in at least one primate, with an overall prevalence of 9.3% (Table 2). Among the isolates analyzed via *fumC* sequencing, two lineages of strains possessed *pet*, with or without co-carriage of ExPEC virulence genes and/or *sepA* (Figure 1). Some of these isolates possessed an IncI1 plasmid, which is characteristic of EAEC strains possessing a virulence plasmid (55). The EAEC-associated gene *pet* is an autotransporter enterotoxin gene that contributes to diarrheal illness, which is predominantly secretory in nature, seen in patients with EAEC (56). Specifically, *pet* was found in the gorillas, spider monkeys, white-faced sakis, and emperor tamarins (Table 3).

Of the three EHEC-associated genes screened, *stx1A* and *etpD* were present in at least one primate (Table 2). *stx2a* was not found in any isolates. The most prevalent EHEC-associated gene, *stx1A* (4.9%), was identified in isolates from the following primate species: De Brazza’s monkeys, spider monkeys, white-faced sakis, and emperor tamarins (Table 3).

Of the two EIEC-associated genes screened, both were present in at least one primate, including *sepA* and *shET2* (Table 2). The most prevalent EIEC-associated gene, *sepA* (10.5%), was identified in isolates from the following primate species: De Brazza’s monkeys, spider monkeys, white-faced sakis, lemurs, and emperor tamarins (Table 3). The EIEC-associated gene *sepA* encodes a serine protease (57). The remaining EIEC-associated gene *shET2* (1.2%) was identified in the De Brazza’s monkeys and emperor tamarins only. The EIEC-associated gene *shET2* encodes an enterotoxin (58). *E. coli* isolated from the gorillas, orangutans, and Geoffroy’s tamarins did not harbor any EIEC-associated genes (Table 3).

### Antimicrobial susceptibility testing

Based on the disk diffusion results, the majority of isolates were susceptible to the seven antibiotics tested (Table 5). However, there were isolates displaying individual or combined decreased antibiotic susceptibility to six of the seven antibiotics tested. Of the antibiotics tested in this study, isolates were most commonly resistant to ampicillin (17.9%) and tetracycline (14.2%). The remaining antibiotics tested had lower occurrence of resistance among the isolates tested (Table 5). Of the eight primate species screened in this study, *E. coli* isolated from feces of De Brazza’s monkeys showed the highest prevalence of ampicillin resistance (33.3%) and *E. coli* isolated from feces of emperor tamarins showed the highest prevalence of tetracycline resistance (41.9%) (Table 5). In contrast, the Geoffroy’s tamarins were fully susceptible to all seven antibiotics tested for the duration of the study. There were nine isolates displaying multidrug resistance, defined as decreased antibiotic susceptibility to >3 classes of antibiotics (59). Five of 27 isolates from white-faced sakis tested were multidrug resistant, which was the most of any primate species tested in this study.

**Table 5:** **Prevalence of antibiotic resistance by primate species (*N*)**.

Antibiotic	Gorilla	Orangutan	De Brazza’s Monkey	Spider Monkey	White-faced Saki	Lemur	Emperor tamarin	Geoffroy’s tamarin
Ampicillin	10.5% (2)	11.1% (3)	33.3% (4)	5.9% (2)	29.6% (8)	0.0% (0)	32.3% (10)	0.0% (0)
Gentamycin	0.0% (0)	0.0% (0)	8.3% (1)	0.0% (0)	0.0% (0)	0.0% (0)	0.0% (0)	0.0% (0)
Nalidixic acid	0.0% (0)	0.0% (0)	0.0% (0)	0.0% (0)	0.0% (0)	0.0% (0)	0.0% (0)	0.0% (0)
Streptomycin	0.0% (0)	0.0% (0)	0.0% (0)	5.9% (2)	18.5% (5)	0.0% (0)	6.5% (2)	0.0% (0)
Tetracycline	0.0% (0)	0.0% (0)	8.3% (1)	8.8% (3)	22.2% (6)	0.0% (0)	41.9% (13)	0.0% (0)
Sulfisoxazole	5.3% (1)	3.7% (1)	0.0% (0)	5.9% (2)	22.2% (6)	20.0% (1)	6.5% (2)	0.0% (0)
Trimethoprim	0.0% (0)	3.7% (1)	0.0% (0)	5.9% (2)	11.1% (3)	0.0% (0)	6.5% (2)	0.0% (0)

## Discussion

For this study, we aimed to determine the prevalence of pathogenic *E. coli* in a captive population of NHPs, to determine the genetic relatedness of *E. coli* over time and between co-habitating (i.e., same primate complex) animal groups, to examine the antimicrobial susceptibility profiles of *E. coli* isolates, and to assess the potential link between the zoo’s history of diarrhea with pathogenic *E. coli* presence*. E. coli* was the organism most suited for the purposes of this study, as it is easily cultured, has been previously associated with diarrheal illness in captive primate populations, and enables assessment of genetic relatedness and microbial gene flow. Initially, we used a phylogenetic typing method to classify the *E. coli* isolates into four groups (A, B1, B2, and D) (37). Of the four phylogenetic groups, B1 is the group most commonly associated with intestinal disease, while A is the group often associated with commensal strains (37). Additionally, previous research indicates that the majority of virulent extraintestinal strains belong to group B2 (37). Virulent extraintestinal strains also belong to phylogenetic group D, but to a lesser degree (37). Our findings suggest that NHPs carry *E. coli* belonging to multiple phylogenetic types, and possibly multiple pathotypes, but whether or not these pathotypes are associated with disease in these animals is unknown at this point.

Genes associated with all aforementioned pathotypes were present among isolates in this NHP collection, but ExPEC-associated genes were the most prevalent pathotype-associated genes identified. ExPEC is best known for its role in causing urinary tract infections (UTIs) and sepsis in human beings (60, 61). However, it is unknown whether or not ExPEC causes disease in NHPs. In terms of UTI pathogenesis, some evidence suggests that monkeys are not suitable hosts for experimental infection models, as they do not appear to develop UTIs naturally from human ExPEC strains (60). Throughout the study period, none of the subjects exhibited any clinical signs suggesting the presence of UTIs or associated sepsis amongst the primate collection and therefore it is more likely that these isolates were a component of the normal GI flora of these animals.

Over the course of this study, all primate species examined, aside from the orangutans, contained at least one isolate testing positive for at least one of the diarrheagenic *E. coli* pathotypes screened, including EPEC, ETEC, EAEC, EHEC, and EIEC. Of these, EPEC-associated genes were the most prevalent. EPEC is a major problem in developing countries because it is a major cause of infant diarrhea, which is sometimes fatal (62). While no causation was established, it is certainly plausible that EPEC could play a role in the intermittent diarrhea seen in this primate population. It is likely that EPEC exhibits different effects on different animals, with some animals acting as asymptomatic carriers and others susceptible to diarrhea (3, 6, 7, 29).

**Figure 2:**
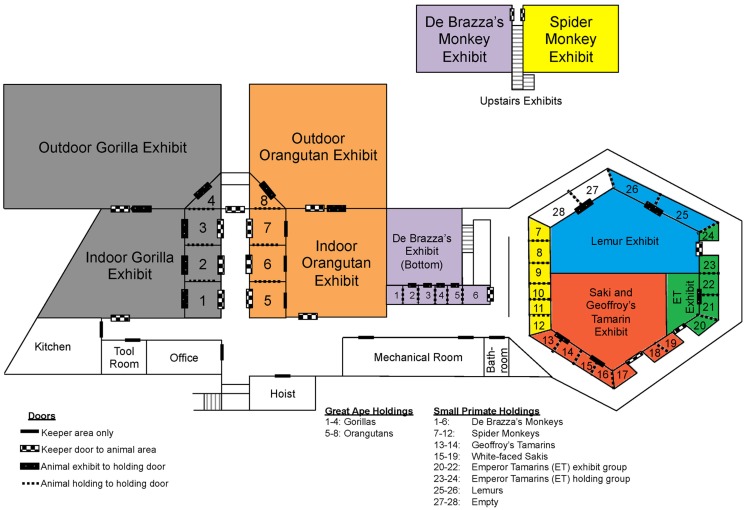
**Map of Como Zoo’s primate enclosure locations, including those in holding and on-exhibit**. Species are colored as follows: gray = gorilla, orange = orangutan, purple = De Brazza’s monkey, yellow = spider monkey, blue = lemur, green = Emperor tamarin, and red = white-faced saki and Geoffroy’s tamarin.

As previously mentioned, we tested each *E. coli* isolate for susceptibility toward the following seven antibiotics: ampicillin, gentamicin, nalidixic acid, streptomycin, tetracycline, sulfisoxazole, and trimethoprim. Past antibiotic treatments attempted at the zoo to treat diarrheal illness have included ampicillin, tetracycline, erythromycin, azithromycin, enrofloxacin, and sulfasalazine. Recently, antibiotics were rarely used in the white-faced saki and emperor tamarin populations, yet we observed a higher percentage of resistance among the *E. coli* isolates obtained from these two primate species (Table 5). In fact, neither the emperor tamarins nor the white-faced sakis were treated with ampicillin, yet *E. coli* isolated from the emperor tamarins showed the highest prevalence of ampicillin resistance (32.3%), and *E. coli* isolated from the white-faced sakis showed the third highest prevalence of antibiotic resistance (29.6%). Along these same lines, the emperor tamarins were never treated with tetracycline, yet *E. coli* isolated from the emperor tamarins showed the highest prevalence of tetracycline resistance (41.9%) (Table 5). The fact that tetracycline resistance and ampicillin resistance were high in the white-faced sakis and emperor tamarins, while antibiotics were used infrequently, could suggest that resistance genes carried by the obtained *E. coli* isolates were not acquired in response to selective pressures. Another plausible explanation is unknown co-selection may be occurring through use of another antibiotic or antimicrobial agent. Also, ampicillin has been used historically at the zoo to treat physical injuries, which are often times incurred by the males. Specifically, ampicillin has been the treatment of choice for the gorillas, orangutans, and spider monkeys. The proximity of the spider monkey holding enclosure to the white-faced sakis and emperor tamarins could be a factor in bacterial transmission, including isolates resistant to ampicillin, as the same zoo staff member typically cleans these three enclosures on a given day (Figure 2). Overall, the rates of resistance observed in this study were much lower than those observed in a previous study examining Snub-nosed monkeys at zoos in China (63). This could be attributed to numerous factors, including geographical location, zoo practices, and primate species examined.

In order to determine the genetic relatedness of *E. coli* over time and between co-habitating (i.e., same primate complex) animal groups, sequence-based typing was performed on a subset of the isolates using the *fumC* gene. The results of the sequence-based typing indicated that *E. coli* strains of similar phylogenetic backgrounds, harboring similar sets of virulence factors, are found in multiple animals and species throughout the primate complex at this zoo (Figure 1). This is reflected in the *fumC* sequence typing, where isolates often belonged to the same sequence types and possessed similar virulence gene repertoires and plasmid types (Figure 1). Specifically, common isolates were found between spider monkeys and white-faced sakis, and between gorillas and orangutans, among others.

One important factor to consider when considering potential routes of bacterial transmission among the primates housed at the Como Zoo’s primate complex is the layout of primate holding and the system being employed at the time of this study for washing enclosures, feeding, among other keeper-related tasks. In an effort to visualize the potential routes of bacterial transmission from different animal groups (i.e., primate species), we constructed a detailed map of the primate complex showing the housing locations of all animal groups (i.e., primate species), as well as the locations of doors and primate keeper areas (Figure 2). The map provides perspective, as it allows for the visualization of where each primate species is housed in relation to the others, including those housed together, in close proximity, and at opposite sides of the complex.

As previously mentioned, results of the sequence-based typing showed that isolates with similar genetic backgrounds were found between spider monkeys and white-faced sakis, and between gorillas and orangutans, among others. As is illustrated in Figure 2, the spider monkeys and white-faced sakis enclosures were physically separated, as were the gorillas and orangutans enclosures. However, the gorillas and orangutans shared the same cleaning equipment, as they were housed in close proximity to each other. The same is true for the white-faced sakis and emperor tamarins. In addition, the cleaning procedure at the zoo was designed so that enclosures were located in relation to each other throughout the complex, as well as the placement of cleaning equipment (e.g., hoses). This allows for one person to clean all enclosures located in the great ape holdings section of the complex while another person cleans the enclosures located in the small primate holdings section of the complex simultaneously (Figure 2). Thus, on any given day, the same keeper would be responsible for cleaning both the spider monkeys and white-faced sakis enclosures (Figure 2). Due to the cleaning system employed by the zoo, bacterial transmission between animals likely occurred. One probable route of bacterial transmission that could have taken place is via fomites; in this case, water hoses, brushes, brooms, clothing, footwear, as well as other primate keeper accessories and cleaning equipment could have served as fomites. Overall, shared usage of cleaning equipment, along with designating one keeper responsible for cleaning the enclosures of multiple primate species simultaneously could have resulted in the sharing of bacteria between physically separated primates species.

Diarrhea in captive primates has long been a problem in both zoo settings and research colonies, including the Como Zoo (22–28). Diarrhea is a medical concern not only because it is physiologically taxing to the animals, but also because it is an ideal mechanism for disease transmission. This makes it both a public health concern and an animal welfare concern. In this study, we identified a number of *E. coli* isolates containing virulence genes from various pathotypes, and there was evidence of clonal transmission of isolates between animals and over time in the complex. Overall, the manifestation of chronic diarrhea in the Como Zoo’s primate collection is a complex problem whose solution will require regular screening for microbial agents and consideration of environmental causes. The use of *E. coli* as a surrogate for pathogen transmission, in this setting, aids in the identification of approaches that can be used in captive NHP populations to reduce such transmission.

## Conflict of Interest Statement

The authors declare that the research was conducted in the absence of any commercial or financial relationships that could be construed as a potential conflict of interest.

## Supplementary Material

The Supplementary Material for this article can be found online at http://www.frontiersin.org/Journal/10.3389/fvets.2014.00024/abstract
