# Supplementary material for: Longitudinal Characterization of Escherichia coli in Healthy Captive Non-Human Primates
Source: Front Vet Sci. 2014 Nov 17;1:24. doi: 10.3389/fvets.2014.00024 (PMC4668849; doi:10.3389/fvets.2014.00024)
Supplement: Supplementary file 3 [file Image_1.PDF]

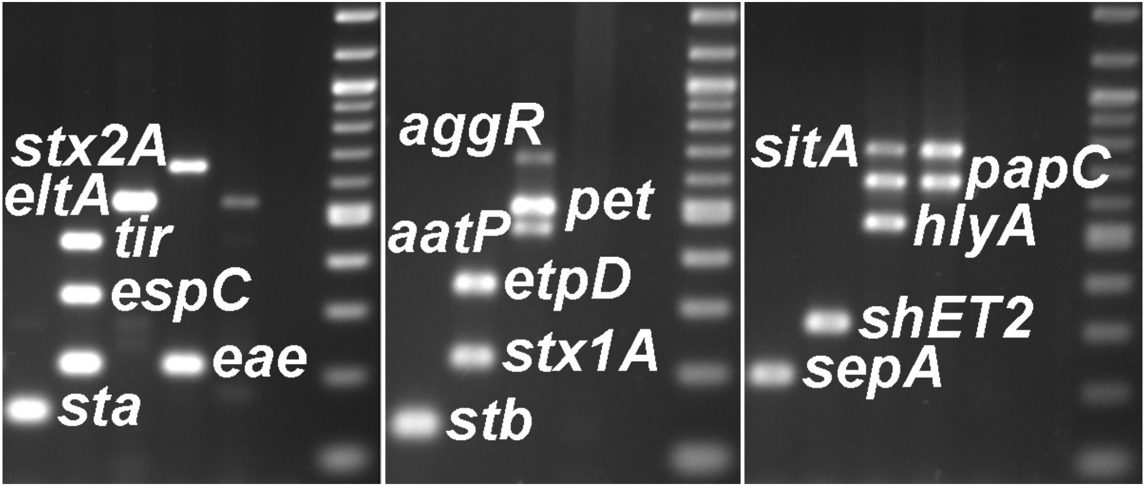

Supplementary Figure 1. Agarose gel electrophoresis of the multiplex panels designed in this study. From left, panels 1, 2, and 3 are shown with positive and negative control strain DNA. Genes corresponding to amplicons are labeled.
